# Supplementary material for: Exploring the growth trait molecular markers in two sheep breeds based on Genome-wide association analysis
Source: PLoS One. 2023 Mar 23;18(3):e0283383. doi: 10.1371/journal.pone.0283383 (PMC10035858; doi:10.1371/journal.pone.0283383)
Supplement: Attachment — Submitted filename: journal requirements.docx [file pone.0283383.s005.docx]

Journal Requirements:

When submitting your revision, we need you to address these additional requirements.

1.Please ensure that your manuscript meets PLOS ONE's style requirements, including those for file naming. The PLOS ONE style templates can be found at

https://journals.plos.org/plosone/s/file?id=wjVg/PLOSOne_formatting_sample_main_body.pdf and <https://journals.plos.org/plosone/s/file?id=ba62/PLOSOne_formatting_sample_title_authors_affiliations.pdf>

　　Answer:We have modified the format of our paper according to the information on the website. Thank you very much for your reminder.

2.Please amend either the abstract on the online submission form (via Edit Submission) or the abstract in the manuscript so that they are identical.

　Answer:We did have some inconsistencies in the submission of our papers. I will revise the paper submitted this time.

3. Thank you for stating the following financial disclosure:“This study was financially supported by grants from the National Natural Science Foundation of China (NO:32060743), Bintuan Science and Technology Program (2022CB001-09) and Efficient mutton sheep breed selection program ( xjnqry-g-2023 )”.

Please state what role the funders took in the study.  If the funders had no role, please state: "The funders had no role in study design, data collection and analysis, decision to publish, or preparation of the manuscript."

If this statement is not correct you must amend it as needed.

Please include this amended Role of Funder statement in your cover letter; we will change the online submission form on your behalf.

　Answer:This study was financially supported by grants from the National Natural Science Foundation of China (NO:32060743),Bintuan Science and Technology Program (2022CB001-09) and Efficient mutton sheep breed selection program ( xjnqry-g-2023 ). The authors thank Xinjiang agricultural area mutton sheep system for providing us with sheep genetic resources population and background information.

4.In your Data Availability statement, you have not specified where the minimal data set underlying the results described in your manuscript can be found. PLOS defines a study's minimal data set as the underlying data used to reach the conclusions drawn in the manuscript and any additional data required to replicate the reported study findings in their entirety. All PLOS journals require that the minimal data set be made fully available. For more information about our data policy, please see <http://journals.plos.org/plosone/s/data-availability>.

Upon re-submitting your revised manuscript, please upload your study’s minimal underlying data set as either Supporting Information files or to a stable, public repository and include the relevant URLs, DOIs, or accession numbers within your revised cover letter. For a list of acceptable repositories, please see [http://journals.plos.org/plosone/s/data-availability#loc-recommended-repositories](http://journals.plos.org/plosone/s/data-availability#loc-recommended-repositories)  Any potentially identifying patient information must be fully anonymized.

Important: If there are ethical or legal restrictions to sharing your data publicly, please explain these restrictions in detail. Please see our guidelines for more information on what we consider unacceptable restrictions to publicly sharing data: http://journals.plos.org/plosone/s/data-availability#loc-unacceptable-data-access-restrictions. Note that it is not acceptable for the authors to be the sole named individuals responsible for ensuring data access.

We will update your Data Availability statement to reflect the information you provide in your cover letter.

Answer:We have uploaded the original data after analysis to the attachment. There are four attachments in total.

5. Please include a copy of Table 6 which you refer to in your text on page 7.

Answer: According to the submission has been modified in the full text.

[Note: HTML markup is below. Please do not edit.]

Reviewers' comments:

Reviewer's Responses to Questions

Comments to the Author

1. Is the manuscript technically sound, and do the data support the conclusions?

The manuscript must describe a technically sound piece of scientific research with data that supports the conclusions. Experiments must have been conducted rigorously, with appropriate controls, replication, and sample sizes. The conclusions must be drawn appropriately based on the data presented.

Reviewer #1: Yes

Reviewer #2: Yes

Reviewer #3: No

2. Has the statistical analysis been performed appropriately and rigorously?

Reviewer #1: Yes

Reviewer #2: Yes

Reviewer #3: No

3. Have the authors made all data underlying the findings in their manuscript fully available?

The [PLOS Data policy](http://www.plosone.org/static/policies.action#sharing) requires authors to make all data underlying the findings described in their manuscript fully available without restriction, with rare exception (please refer to the Data Availability Statement in the manuscript PDF file). The data should be provided as part of the manuscript or its supporting information, or deposited to a public repository. For example, in addition to summary statistics, the data points behind means, medians and variance measures should be available. If there are restrictions on publicly sharing data—e.g. participant privacy or use of data from a third party—those must be specified.

Reviewer #1: Yes

Reviewer #2: No

Reviewer #3: Yes

4. Is the manuscript presented in an intelligible fashion and written in standard English?

PLOS ONE does not copyedit accepted manuscripts, so the language in submitted articles must be clear, correct, and unambiguous. Any typographical or grammatical errors should be corrected at revision, so please note any specific errors here.

Reviewer #1: Yes

Reviewer #2: Yes

Reviewer #3: No

Review Comments to the Author

Please use the space provided to explain your answers to the questions above. You may also include additional comments for the author, including concerns about dual publication, research ethics, or publication ethics. (Please upload your review as an attachment if it exceeds 20,000 characters)

Answer:(1) We selected the growth trait phenotypes of two sheep breeds for genome-wide analysis because the genetic material for growth and development is the same. We conducted a verification experiment on the results of the later period. The result of verification is consistent with that of our GWAS. (2) We analyzed the data by constructing a mixed linear model. Verification is carried out according to statistical requirements.(3) After careful thinking, all the authors sent the original data to the submission system in the form of attached table, and finally appeared in the paper.(4)We have carefully revised the full text, and have found professional embellishments to revise the language.

1. Reviewer #1: In the present manuscript titled "Searching for growth trait molecular markers in two sheep populations based on genome-wide association analysis" by Mirenisa et al., 100 Qira black sheep and 84 German Merino sheep were randomly selected for DNA extraction via jugular vein to prepare Illumine Ovine SNP 50K Bead Chip. Genome-wide association studies (GWAS) were performed on sheep body weight, body height, body length and chest circumference using mixed linear models. 55 SNPs with significant correlation were obtained which were annotated by sheep reference genome and a total of 84 genes were obtained. BMPR1B was selected for population validation, and a correlation between FecB locus and body weight traits was found. This study provides supplementary work for molecular markers of sheep growth traits and has important theoretical significance and reference value for the mining of functional genes of sheep growth traits. This research is well-designed and interesting; however, there are still some concerns, which need to be improved by the authors.
   Please check the grammer seriously, and the whole manuscript has to be proofread by a professional grammer agency.

Answer:The paper was polished by a professional institution. The supporting material has been placed in the corresponding document.

2. Please check the references and unify the format of references.
Answer:References have been modified in accordance with the document format of *Plos one*.

3. The introduction part is too long, I suggested the writer should simplify this part. This part just listed some references.
Answer:Some parts of the introduction have been removed. The deleted part is:In the GWAS study related to animal growth traits, An, *et al.* [2] detected 6 SNPs related to body height and length of Japanese wagyu cattle using Illumina CATTLE HD 770K SNP typing chip. These SNPs were located in or near 11 genes, five of which were novel candidate genes associated with body weight. An, *et al.* [3] used Illumina cattle 770K chip, combined with long-GWAS, single-trait GWAS and multi-trait GWAS, to study the changes of heart size, abdominal circumference, height, body length and tube circumference of Simmental beef cattle at different growth stages. The results showed that 58 significant SNPs were detected by the three models. 21 genes related to body size of Chinese cattle were matched. Through GWAS of backfat thickness in 304 Italian large white pigs, Fontanesi, *et al.*[4] found that *PDE1C*, *CRISP1*, *STAT4* and *STAB1* genes were significantly correlated with backfat thickness. Becker, *et al.* [5] selected 193 boars from the Swiss large white pig population after artificial insemination, and found one QTL locus on chromosome 14 and chromosome 2 for PH1 and carcass length, respectively, and two QTLS on chromosome 10 and chromosome 16 for hindleg length. Martin, *et al.* [6] conducted GWAS analysis of multiple nipples in 1185 alpine dairy goats and 810 Saneng dairy goats from France, and found that there were 17 SNPs with significant chromosome level on chromosome 10, but no SNPs with significant genomic level, which also indicated that multiple nipples were inherited in the form of multiple genes.

4. “A total of 461,528 SNPs were obtained by genotyping.” You only used the SNP 50 chip, you only get 50K SNPs from each individual. The expression of this sentence is not clear.
Answer: It should be 46,871 SNPs. This is my fault in work. I apologize to my esteemed editor.

5. How to explain the data deviation of the QQ plot? You should discuss this question in the discussion part. Your explanation of the QQ plot is not accepted. Please consider carefully the cause of the deviation of the QQ plot.
Answer:If there is deviation, it means that the actual value is deviated from the predicted value. In the GWAS study, if the SNP has a large deviation, it is believed that the deviation of the observed value of this SNP site is caused by the genetic effect of this SNP mutation.

6. The number of sample individual is 184. As we know, the GWAS requires large sample size to ensure the accuracy of result.
Answer:The data we collected from 184 sheep proved to be reliable. All of them were collected personally.

7. “phenol-chloroform method” You need to provide more detailed steps or reference.
Answer:Main steps of DNA extraction by phenol-chloroform method:

1. The animal tissue was placed in a 1.5-ml centrifuge tube and dealcoholized with 75%, 50% alcohol and pure water gradients, respectively. Each of the ladder Degree dehydration time is 5-10 min.

2. Place the tissue in a mortar, add appropriate amount, and rinse the grinding rod.

3. Add the ground tissue fluid to the seal of the heart tube with a pipette gun and put it into the shaker (56℃, 5h).

4. Add an equal volume of Tris saturated phenol (500 μl) and shake well (10 min).

5. Centrifugation: 12,000 rpm, 7 min, 4℃. After centrifugation, it is divided into three layers, the upper layer is DNA, the middle layer is protein, and the lower layer is organic matter.

6. Drain the top liquid and add it to the new centrifuge tube.

7. Tris saturated phenol: chloroform: isoamyl alcohol =25:24:1.

8. Add 450 μl of Tris saturated phenol, chloroform and isoamyl alcohol mixture to centrifuge tube containing supernatant and shake well for 10 min.

9. Centrifugation: 12,000 rpm, 7 min, 4℃.

10. Drain the supernatant and add it to a new centrifuge tube. Add an equal volume of 400 μl mixture of chloroform and isoamyl alcohol (chloroform: isoamyl alcohol = 24:1).

11. Centrifugation: 12,000 rpm, 7 min, 4℃.

12. Absorb the supernatant and add it to the new centrifuge tube. Add 2.5 times of 100% alcohol frozen at -20℃, overnight at -20℃.

13. The sample is taken out and centrifuged at 12,000 rpm, 7 min, 4℃.

14. Discard supernatant, leave white precipitate (DNA), add 75% alcohol frozen at -20℃, 400 μl, blow and dissolve repeatedly the solution.

15. Repeat step 14, 2 times (wash three times with 75% alcohol).

16. DNA extraction is complete.

We have cited reference to DNA extraction [1].

1. Sambrook J, Fritschi EF,Maniatis T.Molecular Cloning: A Laboratory Manual.Cold Spring Harbor Laboratory Press.1989;

8. The GWAS analysis always used enrichment analysis of candidate genes and pathways using GO and KEGG. According to the SNPs how to find these candidate genes? You should provide more details.
Answer:We used TASSEL5.0 software to analyze the SNP chip, and the SNP coordinate data came out for gene annotation. With the Sheep Genome Library (oar_rambouillet_v4.0 ftp://ftp.ncbi.nlm.nih.gov/genomes/all/GCF/002/742/125/GCF_002742125.1_oar_rambouillet_v4.0_genomic.fna.g), we found candidate genes.

9. “The genetic matrix was calculated by KING software and mapped by R language.” You should provide the name of the R package you used.
Answer:The name of the R package is qqMAN.

10. The abstract part needs to be modified. This part should not to specifically mention the analysis software you used.

Answer: We have modified the abstract and deleted the analysis software.

11. In discussion part, you should discuss how to use these candidate genes for molecular markers in sheep genetics and breeding.

Answer:

1. Find relevant marker genes according to breeding objectives.
2. Identify the marker genes and detect the polymorphic loci of the marker genes in the population.
3. The influence of marker genes on marker traits was analyzed.
4. Favorable genotypes were selected according to the analysis results.

2. Reviewer #2: In the present manuscript titled "Searching for growth trait molecular markers in two sheep populations based on genome-wide association analysis" by Mirenisa et al., 100 Qira black sheep and 84 German Merino sheep were randomly selected for DNA extraction via jugular vein to prepare Illumine Ovine SNP 50K Bead Chip. Genome-wide association studies (GWAS) were performed on sheep body weight, body height, body length and chest circumference using mixed linear models. 55 SNPs with significant correlation were obtained which were annotated by sheep reference genome and a total of 84 genes were obtained. BMPR1B was selected for population validation, and a correlation between FecB locus and body weight traits was found. This study provides supplementary work for molecular markers of sheep growth traits and has important theoretical significance and reference value for the mining of functional genes of sheep growth traits. This research is well-designed and interesting; however, there are still some concerns, which need to be improved by the authors.
   Please check the grammer seriously, and the whole manuscript has to be proofread by a professional grammer agency.

Answer:The paper was polished by a professional institution. The supporting material has been placed in the corresponding document.

2. Please check the references and unify the format of references.
Answer:References have been modified in accordance with the document format of *Plos one*.

3. The introduction part is too long, I suggested the writer should simplify this part. This part just listed some references.
Answer:Some parts of the introduction have been removed. The deleted part is:In the GWAS study related to animal growth traits, An, *et al.* [2] detected 6 SNPs related to body height and length of Japanese wagyu cattle using Illumina CATTLE HD 770K SNP typing chip. These SNPs were located in or near 11 genes, five of which were novel candidate genes associated with body weight. An, *et al.* [3] used Illumina cattle 770K chip, combined with long-GWAS, single-trait GWAS and multi-trait GWAS, to study the changes of heart size, abdominal circumference, height, body length and tube circumference of Simmental beef cattle at different growth stages. The results showed that 58 significant SNPs were detected by the three models. 21 genes related to body size of Chinese cattle were matched. Through GWAS of backfat thickness in 304 Italian large white pigs, Fontanesi, *et al.*[4] found that *PDE1C*, *CRISP1*, *STAT4* and *STAB1* genes were significantly correlated with backfat thickness. Becker, *et al.* [5] selected 193 boars from the Swiss large white pig population after artificial insemination, and found one QTL locus on chromosome 14 and chromosome 2 for PH1 and carcass length, respectively, and two QTLS on chromosome 10 and chromosome 16 for hindleg length. Martin, *et al.* [6] conducted GWAS analysis of multiple nipples in 1185 alpine dairy goats and 810 Saneng dairy goats from France, and found that there were 17 SNPs with significant chromosome level on chromosome 10, but no SNPs with significant genomic level, which also indicated that multiple nipples were inherited in the form of multiple genes.

4. “A total of 461,528 SNPs were obtained by genotyping.” You only used the SNP 50 chip, you only get 50K SNPs from each individual. The expression of this sentence is not clear.
Answer: It should be 46,871 SNPs. This is my fault in work. I apologize to my esteemed editor.

5. How to explain the data deviation of the QQ plot? You should discuss this question in the discussion part. Your explanation of the QQ plot is not accepted. Please consider carefully the cause of the deviation of the QQ plot.
Answer:If there is deviation, it means that the actual value is deviated from the predicted value. In the GWAS study, if the SNP has a large deviation, it is believed that the deviation of the observed value of this SNP site is caused by the genetic effect of this SNP mutation.

6. The number of sample individual is 184. As we know, the GWAS requires large sample size to ensure the accuracy of result.
Answer:The data we collected from 184 sheep proved to be reliable. All of them were collected personally.

7. “phenol-chloroform method” You need to provide more detailed steps or reference.
Answer:Main steps of DNA extraction by phenol-chloroform method:

1. The animal tissue was placed in a 1.5-ml centrifuge tube and dealcoholized with 75%, 50% alcohol and pure water gradients, respectively. Each of the ladder Degree dehydration time is 5-10 min.

2. Place the tissue in a mortar, add appropriate amount, and rinse the grinding rod.

3. Add the ground tissue fluid to the seal of the heart tube with a pipette gun and put it into the shaker (56℃, 5h).

4. Add an equal volume of Tris saturated phenol (500 μl) and shake well (10 min).

5. Centrifugation: 12,000 rpm, 7 min, 4℃. After centrifugation, it is divided into three layers, the upper layer is DNA, the middle layer is protein, and the lower layer is organic matter.

6. Drain the top liquid and add it to the new centrifuge tube.

7. Tris saturated phenol: chloroform: isoamyl alcohol =25:24:1.

8. Add 450 μl of Tris saturated phenol, chloroform and isoamyl alcohol mixture to centrifuge tube containing supernatant and shake well for 10 min.

9. Centrifugation: 12,000 rpm, 7 min, 4℃.

10. Drain the supernatant and add it to a new centrifuge tube. Add an equal volume of 400 μl mixture of chloroform and isoamyl alcohol (chloroform: isoamyl alcohol = 24:1).

11. Centrifugation: 12,000 rpm, 7 min, 4℃.

12. Absorb the supernatant and add it to the new centrifuge tube. Add 2.5 times of 100% alcohol frozen at -20℃, overnight at -20℃.

13. The sample is taken out and centrifuged at 12,000 rpm, 7 min, 4℃.

14. Discard supernatant, leave white precipitate (DNA), add 75% alcohol frozen at -20℃, 400 μl, blow and dissolve repeatedly the solution.

15. Repeat step 14, 2 times (wash three times with 75% alcohol).

16. DNA extraction is complete.

We have cited reference to DNA extraction [1].

1. Sambrook J, Fritschi EF,Maniatis T.Molecular Cloning: A Laboratory Manual.Cold Spring Harbor Laboratory Press.1989;

8. The GWAS analysis always used enrichment analysis of candidate genes and pathways using GO and KEGG. According to the SNPs how to find these candidate genes? You should provide more details.
Answer:We used TASSEL5.0 software to analyze the SNP chip, and the SNP coordinate data came out for gene annotation. With the Sheep Genome Library (oar_rambouillet_v4.0 ftp://ftp.ncbi.nlm.nih.gov/genomes/all/GCF/002/742/125/GCF_002742125.1_oar_rambouillet_v4.0_genomic.fna.g), we found candidate genes.

9. “The genetic matrix was calculated by KING software and mapped by R language.” You should provide the name of the R package you used.
Answer:The name of the R package is qqMAN.

10. The abstract part needs to be modified. This part should not to specifically mention the analysis software you used.

Answer: We have modified the abstract and deleted the analysis software.

12. In discussion part, you should discuss how to use these candidate genes for molecular markers in sheep genetics and breeding.

Answer:

1. Find relevant marker genes according to breeding objectives.
2. Identify the marker genes and detect the polymorphic loci of the marker genes in the population.
3. The influence of marker genes on marker traits was analyzed.
4. Favorable genotypes were selected according to the analysis results.

1. Reviewer #3: The authors presented a manuscript on growth trait molecular markers in two sheep populations based on genome-wide association analysis. The topic of the investigation is interesting but there are several points of concern that make the paper not acceptable for the publication.
   General comments:
   -why the authors carried out their study using Qira black and German Merino breeds? Is there any etnological reason? This is a main problem. Without furnish a scientific reason about it, the the work seems unjustified, making the manuscript evaluation quite difficult.

Answer:The genetic law of growth and development traits in different sheep populations are the same. Growth and development traits are controlled by micro-effect polygenes. The body weight, body height, body length and chest circumference in our study are part of growth and development traits; and it is also controlled by micro-effect polygenes. When the two populations are analyzed together, growth and development traits can be more accurately and widely found in terms of genetic diversity.

2. -in the material and methods section several information are missing. Please remeber that this section has to furnish all the information to make the investigation repeatable.

Answer:We added the detailed information after the modification, to make the investigation repeatable.

3. -I am not able to understand why the authors genotyped the animals for polymorphisms at the BMPRIB gene… This is not specified in the aim of the work.
   Answer:FecB is a molecular marker of *BMPRIB*, which was found by genome-wide association analysis (P=1.29×10^-7^, in weight trait). The aim of genotyping the animals for polymorphisms was to further verify the accuracy of genome-wide association analysis. We selected the results of this study for verification, and the verification result showed that there was a mutation site in *BMPR1B* gene, A > G mutation, and there were three genotypes: AA, AG and GG, which were consistent with our results after population verification.

Specific comments:

1. -please if possibile include the line numbers that make the reviewer’s work easier.

Answer:All the contents of the paper have been numbered.

2. -keywords: it is better to replace character with trait

Answer:The key words of the paper have been revised according to the opinion of the editor.

3. -in the introduction section several sentences need references. Moreover the introduction section has to be shorted, avoiding information about cattle and focusing your attention on sheep. The list of the available SNPs chip in different specie is not updated.

Answer:Some information about cattle and quotations in the introduction section have been deleted. The introduction has been simplified.

Material and methods
1.-please supply information about the Qira black sheep breed, i.e. present census, number of farms, geographic distribution, productive purpose, under conservation plan? Were the animals sampled only females? In the statistical model is reported also “gender” and so I am confused about it.

Answer:Qira black sheep breed has a long history, about 120 years of history. After long-term careful cultivation by local herdsmen, it has formed a local excellent sheep breed with stable genetic performance, large body, robust and adapted to arid desert ecological environment. It is mainly distributed in the Nuer ranch of Qira County, Hotan region, and there are only about 4,000 sheep left, which is a local protected sheep breed. It is only distributed in Qira County, Hotan region, mainly concentrated in Nuer ranch and the surrounding towns. The sheep breed has delicious meat and is rich in mineral elements, especially selenium, and the local government has made a breeding conservation plan. The animals sampled were all female, which reduced the influence of male and female factors on the growth and development of individual animals. We have made some modifications to the model of the species.

2.-the authors reported “84 unrelated sheep” and few lines later they stated “no genealogical information”. The two statements are in opposition….. Please clarify. Same for “standardized feeding”: this is not adequate for a scientific paper. Please specify the feeding supplied to the animals.
Answer:Sheep breeding experts in the southern region of Xinjiang, China, have standardized the forage materials for sheep forage breeding. The aim of adding 84 German Merino sheep is to better deal with genetic diversity errors. In this paper, we checked the samples and made modifications.

3.-please specify at what age the morphological traits were measured.

Answer:A total of 84 German Merino sheep (from Kezhou Breeding Farm) and 100 Qira black sheep (from Qira County Tianjin Aoqun Livestock Breeding Sheep Farm) among 1,200 ewes were genotyped using the Illumina 50K SNP panel for 54,241 markers. The body height, body length, weight, and chest circumference of the experimental animals were measured using electronic scales, measuring rods, and tape measures.

4. -I am not able to understand what the authors mean with “experimental animals for population validation were 89 Qira black….”.

Answer:We obtained 55 significant SNPs loci, among which 11 loci reached an extremely significant level. Population validation of *BMPR1B* confirmed our study results.Because after GWAS analysis, we verified the candidate genes and collected 89 Qira black sheep for population verification.

5. -please supply a reference for DNA extraction protocol.
   Answer:In the materials and methods, we have cited a reference [1] to DNA extraction.
1. Sambrook J, Fritschi EF,Maniatis T.Molecular Cloning: A Laboratory Manual.Cold Spring Harbor Laboratory Press.1989;

Results
1.The authors genotyped all the animals using the Illumina Ovine 50K. In the first line of results they stated “a total of 461,528 SNPs were obtained..”… How is possible?

Answer: It should be 46,871 SNPs. This is my fault in work. I apologize to my esteemed editor.

2.The main part of results is not about GWAS but about chromosome wide. This aspect has to be appropriately highlighted.

Answer: It has been adjusted according to the pattern and requirements.The results are presented in the form of tables and figures.

3.A true discussion section is missing. The main part of the discussion is a bibliographic review on gene onthology. The authors have to compare in this section the results of their study with the available literature on the same topic.

Answer:Discussions have been held as requested.The false positive phenomenon of GWAS analysis was mainly due to population stratification, which eventually gave rise to multiple SNPs loci associated with the concerned traits in the GWAS analysis results [1]. To reduce these false-positive results, population stratification and relatedness among individuals were fully considered. We used PCA and breed effect to solve the population stratification phenomenon. As can be seen from the QQ-plot, there was no population stratification phenomenon in the corrected population, and the GWAS analysis results based on the MLM were relatively reliable. Additionally, the TASSEL 5.0 software provides a kinship matrix, two correction a group the genetic relationship between the two individuals[Please check this as the meaning is not clear.], to improve the effectiveness of the GWAS analysis. In this study, we performed a GWAS on the Qira black sheep and German Merino sheep via genotyping data by using a medium-density chip containing 46,871 SNPs. We detected 55 SNPs that were significantly associated with growth traits. From the genomic screening information, we found over 10% of the genes to be located in the sheep chromosomes 1, 2, 6, and 12. Among them, 10 loci were located on chromosome 1 (OAR1_223367286.1, S06270.1, S33133.1, etc.). There were 6 SNPs loci on chromosome 2, 6, and 12 (OAR2_179242768.1, OAR6_111740006.1, OAR12_60622021.1, etc.) We obtained a total of 84 genes through gene annotation. Based on gene enrichment analysis, we can preliminarily infer that these loci were important molecular markers that affected the growth and meat production traits of sheep.

Zhang et al. [2] had obtained 36 significant SNPs for seven traits, while we obtained 55 significant SNPs for 4 traits (body weight, body height, body length, and chest circumference), which enriched the SNPs related to sheep growth and development. [Furthermore, Jiang et al. [3] obtained 5 SNPs related to body height and 4 SNPs related to chest circumference in Hu sheep,](https://pubmed.ncbi.nlm.nih.gov/?term=Jiang+J&cauthor_id=34093644) whereas Hawlader et al. [4] obtained 39 SNPS related to body height in Australian Merino sheep. However, we identified 9 and 13 SNPs correlated with body height and chest circumference in this study, respectively. Since we had selected different germplasm resources for this study, we obtained the results at different loci. Although some of the annotated genes have already been previously reported, we found genes related to growth and development traits, including *POMK*, *MEIS1*, *CROT*, *BMPR1B*, *FABP5*, *SPATA17*, etc. Based on the NCBI and Gene Cards database annotations, we found that among the meat quality traits, genes related to growth traits mainly included *POMK*, *BMPR1B*, *FABP5*, *RPL17*, *EFNA5*, *NELL1* and *PPARGC1A*.

1. Lander E, Kruglyak L. Genetic dissection of complex traits: guidelines for interpreting and reporting linkage results. Nat Genet. 1995;11:241-247.<http://doi.org/>10.1038/ng1195-241 PMID: 7581446.
2. Zhang L, Liu J, Zhao F, Ren H, Xu L ,Lu J, et al. Genome-Wide Association Studies for Growth and Meat Production Traits in Sheep. PLoS ONE.2013;8: e66569.<http://doi.org/>10.1371/journal.pone.0066569 PMID: 23825544
3. Jiang J, Cao Y, Shan H, Wu J, Song X, Jiang Y. The GWAS Analysis of Body Size and Population Verification of Related SNPs in Hu Sheep. Front Genet. 202;12:642552. <http://doi.org/>10.3389/fgene.2021.642552 PMID: 34093644
4. Al-Mamun HA, Kwan P, Clark SA, Ferdosi MH, Tellam R, Gondro C. Genome-wide association study of body weight in Australian Merino sheep reveals an orthologous region on OAR6 to human and bovine genomic regions affecting height and weight. Genet Sel Evol. 2015;47(1):66.<http://doi.org/>10.1186/s12711-015-0142-4 PMID:26272623

6. PLOS authors have the option to publish the peer review history of their article ([what does this mean?](https://journals.plos.org/plosone/s/editorial-and-peer-review-process#loc-peer-review-history)). If published, this will include your full peer review and any attached files.


If you choose “no”, your identity will remain anonymous but your review may still be made public.


Do you want your identity to be public for this peer review? For information about this choice, including consent withdrawal, please see our [Privacy Policy](https://www.plos.org/privacy-policy).

Reviewer #1: No

Reviewer #2: No

Reviewer #3: No

Answer: NO，Respect the privacy of authors and reviewers.

[NOTE: If reviewer comments were submitted as an attachment file, they will be attached to this email and accessible via the submission site. Please log into your account, locate the manuscript record, and check for the action link "View Attachments". If this link does not appear, there are no attachment files.]

While revising your submission, please upload your figure files to the Preflight Analysis and Conversion Engine (PACE) digital diagnostic tool, <https://pacev2.apexcovantage.com/>. PACE helps ensure that figures meet PLOS requirements. To use PACE, you must first register as a user. Registration is free. Then, login and navigate to the UPLOAD tab, where you will find detailed instructions on how to use the tool. If you encounter any issues or have any questions when using PACE, please email PLOS at [figures@plos.org](mailto:figures@plos.org). Please note that Supporting Information files do not need this step.
