# Supplementary material for: Exploring the growth trait molecular markers in two sheep breeds based on Genome-wide association analysis
Source: PLoS One. 2023 Mar 23;18(3):e0283383. doi: 10.1371/journal.pone.0283383 (PMC10035858; doi:10.1371/journal.pone.0283383)
Supplement: Attachment — Submitted filename: review comments.docx [file pone.0283383.s006.docx]

For this study, The study obtained molecular markers related to sheep growth traits, which formed the basis of molecular breeding. In this study, randomly selected 100 Qira black sheep and 84 German Merino sheep for DNA extraction via the jugular vein to prepare the Illumine Ovine SNP 50K Bead Chip. Quality control criteria were: rejection detection rate < 90% and minimum allele frequency (MAF) < 5%. Then, The study performed genome-wide association studies (GWAS) on sheep body weight, body height, body length, and chest circumference using mixed linear models. After getting 55 SNPs with significant correlation, they were annotated by reference genome of Ovis aries (Oar_v4.0) and it obtained a total of 84 genes (BMPR1B, HSD17B3, TMEM63C, etc.). The study selected BMPR1B for population validation and found a correlation between the FecB locus and body weight traits. Therefore, this study not only supplements the existing knowledge of molecular markers of sheep growth traits, but also has important theoretical significance and reference value for the mining of functional genes of sheep growth traits.Therefore, I agree to accept the current revision.

**Comments to the Author**

I have no other questions.
I agree to accept the current revision.
