# Supplementary material for: Exploring the growth trait molecular markers in two sheep breeds based on Genome-wide association analysis
Source: PLoS One. 2023 Mar 23;18(3):e0283383. doi: 10.1371/journal.pone.0283383 (PMC10035858; doi:10.1371/journal.pone.0283383)
Supplement: Attachment — Submitted filename: Response to Reviewers.docx [file pone.0283383.s007.docx]

Review Comments to the Author

1. -title: the two populations studied are populations or breeds? It is important to take in consideration this aspect. The term population is usually used when no herd book is available. On the contrary it is better to use the term breed.

Answer:The two populations studied are varieties, and we have used the term variety in this thesis in accordance with the editor's comments.

2. -line 29:"We obtained" is not adequate . May be "we identified" . Please check .

Answer:Following the editor's advice, we proceeded to check the paragraph. Change we obtained ("we obtained") to we identified ("we identified").

3. -line 32:"via the jugular vein” is for the blood collection and not for the DNA extraction. Moreover, what means "to prepare the Illumine Ovine SNP 50K Bead Chip"? May be "to genotype by using the Illumina Ovine SNP 50K Bead Chip” .

Answer:Line 32, through the jugular vein does to collect blood, here write DNA is my revision on the carelessness, this part has been corrected."to prepare the Illumine Ovine SNP 50K Bead Chip"Changed to"to genotype by using the Illumina Ovine SNP 50K Bead Chip” .

4. -line 32:please replace "quality control criteria were” with "quality control criteria for statistical analysis were”

Answer:According to the editor's comments in line 32 of the paper "quality control criteria were”Modified to"quality control criteria for statistical analysis were”

5. -line 36:please use italic style for "Ovis aries”and add"genome”soon after Ovis aries.

Answer:Line 36 of"Ovis aries”use italic style，and add"genome”soon after Ovis aries.

6. -line 37:"we obtained a total of 84 genes".this sentence is not complete.84 genes for what? Associated to productive traits? Please specify it.

Answer:Here we obtained a total of 84 candidate genes associated with production traits.

7. -line 46 and following lines; again I am not able to understand why the authors are referring to researches on deseases(in human)when there is a lot of literature available on the use of GWAS in animal genetics. My suggestion is to delete this part that it is out of contest.

Answer:We have removed the section on human GWAS as per the editor's comments.

8. -line 58:please replace "the sheep 50 K" with "the ovine 50 K".

Answer:According to the editor's comments in line 58 of the paper "the sheep 50 K"Modified to"the ovine 50 K"。

9. -line 107, please specify model and company for biophotometer.

Answer:The type of biological photometer is：DS-11，company　is：DeNovix, USA

10. -line 120:please supply a reference for the KING software

Answer:Reference for added software

11. -line 123Ｃplease replace“GWAS analysis of four body weight traits in 184 sheep was performed using Illumina OvineSNP50 microarray" with "GWAS analysis of four body weight traits in 184 sheep was performed using genotypes obtained from Illumina OvineSNP50 microarray".

Answer:In accordance with the suggestions made by the editorial faculty in the paper“GWAS analysis of four body weight traits in 184 sheep was performed using Illumina OvineSNP50 microarray"Part has been changed to"GWAS analysis of four body weight traits in 184 sheep was performed using genotypes obtained from Illumina OvineSNP50 microarray"

12. -line 137:please check the use of"P” with capital letter and without in the same line.

Answer:Change the "P" in this paragraph and throughout the paper to italics.

13. -line 139:please avoid paragraph so short. It could be better to merge this one with the previous one.

Answer:As suggested by the editor, this and the previous paragraph have been combined.

14. -line 144: please supply a reference for Primer Premier 6.0 software.

Answer:Reference for added software

15. -line 148, please specify the country for Shanghai Shenggong Bioengineering Company.

Answer:The country mentioned in line 148, to which Shanghai Biotech belongs, is China and has been added in the paper.

16. -table 1:please replace the title of the first column with "Gene” .

Answer:in the title of Table I "gene” Changed to"Gene”

17. -line 151:about the PCR conditions:please mention the final/stock concentrations of each reagent, without which it's meaningless.

Answer:PCR reagent concentration has been added

18. -line 158, please supply a reference for DNASTAR

Answer:PCR reagent concentration has been added

19. -line 159:please supply a reference for SPSS 26.0 software

Answer:Software reference has been added.

20. -line 160 please replace coma with dot.

Answer:Modified

21. -line 176:German-American sheep or German Merino sheep? Please clarify.

Answer:Here is the German Merino sheep, which has also been revised in the paper. This is an oversight in my work, so please apologize to the teacher.

22. -line 184: there are typos in this line, please check.

Answer:Modified

23. -line 190: the sentence"with significant GWAS analysis results"is not correct. May be "with significant associations with productive traits obtained in the GWAS analysis”

Answer:This section has been deleted：with significant GWAS analysis results，and add with significant associations with productive traits obtained in the GWAS analysis。

24. -line 213, Qira with capital letter.

Table 3: please use the italic style for all the gene acronyms (check in all the manuscript long).

Answer:The corresponding part of the paper has been revised according to the editor's comments.

25. -line 223:what is "Cele black sheep"??

Answer:Here should be "Qira black sheep"，This has been fixed.

26. -line 227, please replace “amplified” with "amplified region of”

Answer:Follow the editor's advice“amplified”Changed to"amplified region of”

27. -line 230, gene acronym in italic style as in the following line.

Answer:Gene names have been changed to italics

28. -line 239:in my opinion this sentence is not complete:"As can be seen from Table 4, the BMPR1B gene g.431965A>G in the Qira black sheep”

Answer:According to what the editor said we check the paper, this part is indeed incomplete, and we have revised this paragraph.

29. -line 241:was the significance level the same among the different genotypes? In the text seems to be 0.05 but in the Table 4 there are two different levels(a and b). Please clarify.

Answer:Not the same, this part has been modified.

30. -line 379:it could be better to replace "genome microchips"with "SNPs data” Finally try to improve the conclusion section with a message to take at home for the breeders: try to explain the transferability of your results and in what way these results can be important in animal breeding of the studied breeds.

Answer: The conclusion has been partially revised
